# Fe^III^ in the high-spin state in di­methyl­ammonium bis­[3-eth­oxy­salicyl­aldehyde thio­semi­car­ba­zon­ato(2–)-κ^3^ *O* ^2^,*N* ^1^,*S*]ferrate(III)

**DOI:** 10.1107/S2053229622011597

**Published:** 2023-01-01

**Authors:** Robyn E. Powell, Martin R. Lees, Graham J. Tizzard, Simon J. Coles, Qingchun Yuan, Petra J. van Koningsbruggen

**Affiliations:** a College of Engineering and Physical Sciences, School of Infrastructure and Sustainable Engineering, Department of Chemical Engineering and Applied Chemistry, Aston University, Aston Triangle, Birmingham, West Midlands, B4 7ET, United Kingdom; b Department of Physics, University of Warwick, Coventry, CV4 7AL, United Kingdom; c National Crystallography Service, Chemistry, University of Southampton, Southampton, SO17 1BJ, United Kingdom; d Energy and Bioproducts Research Institute, College of Engineering and Physical Sciences, Aston University, Birmingham, B4 7ET, United Kingdom; University of Monash, Australia

**Keywords:** Fe^III^ com­plex, high-spin, crystal structure, thio­semicarbazone, salicyl­aldehyde, magnetic susceptibility

## Abstract

The synthesis and crystal structure (100 K) of the title com­pound, [(CH_3_)_2_NH_2_][Fe(C_10_H_11_O_2_N_3_S)_2_], are reported. The asymmetric unit consists of an octa­hedral [Fe^III^(*L*)_2_]^−^ fragment, where *L* ^2−^ is 3-eth­oxy­salicyl­aldehyde thio­semi­car­ba­zon­ate(2−), and a di­methyl­ammonium cation. Each *L* ^2−^ ligand binds with the thiol­ate S, the imine N and the phenolate O atoms as donors, resulting in an Fe^III^S_2_N_2_O_2_ chromophore. The ligands are orientated in two perpendicular planes, with the O and S atoms in *cis* positions, and mutually *trans* N atoms. The Fe^III^ ion is in the high-spin state at 100 K. The variable-tem­per­ature magnetic susceptibility measurements (5–320 K) are consistent with the presence of a high-spin Fe^III^ ion with *D* = 0.83 (1) cm^−1^ and *g* = 2.

## Abstract 2

The Fe^III^S_2_N_2_O_2_ chromophore contains two *O*,*N*,*S*-donating dianionic ligands in perpendicular planes, with the O and S atoms in *cis* and the N atoms in *trans* positions. The Fe^III^ ion is in the high-spin state at 100 K. The variable-tem­per­ature magnetic susceptibility measurements (5–320 K) are consistent with the presence of a high-spin *S* = 5/2 Fe^III^ ion.

## Introduction

The continuing research and development of switchable mag­netic, optical and/or photomagnetic materials seeks to provide solutions for the societal desire towards more advanced electronic devices (*e.g.* larger data storage capacity and faster data processing) and their miniaturization by offering industry novel magnetic materials that can be implemented in electronic devices for information storage and as displays (Létard *et al.*, 2004 ▸; Gütlich *et al.*, 2004 ▸; Gütlich & Goodwin, 2004 ▸; van Koningsbruggen *et al.* 2004 ▸; Halcrow, 2013 ▸; Molnár *et al.*, 2018 ▸; Senthil Kumar *et al.*, 2017 ▸; Rubio-Giménez *et al.*, 2019 ▸; Tissot *et al.*, 2019 ▸; Karuppannan *et al.*, 2021 ▸). Spin-crossover materials have attractive physical properties that make them suitable candidates for fulfilling these requirements. Such com­pounds exhibiting a tem­per­ature-dependent crossover between electronic states having a different magnetic moment were first discovered for iron(III) tris­(di­thio­carbamates) (Cambi & Szegö, 1931 ▸, 1933 ▸). Since then, two main families of Fe^III^ spin-crossover systems have been extensively studied, *i.e.* those containing ligands sporting chalcogen donor atoms and those based on multidentate *N*,*O*-donating Schiff base-type ligands (van Koningsbruggen *et al.*, 2004 ▸; Harding *et al.*, 2016 ▸). It has been found that the magnetic inter­conversion between the low-spin (*S* = 1/2) and high-spin (*S* = 5/2) state in Fe^III^ systems can be triggered by a change in tem­per­ature or pressure, or by light irradiation (Hayami *et al.*, 2000 ▸, 2009 ▸; van Koningsbruggen *et al.*, 2004 ▸; Harding *et al.*, 2016 ▸).

The generation of Fe^III^ spin-crossover behaviour using par­ticular salicyl­aldehyde thio­semicarbazone derivatives has been extensively studied by several research groups (van Koningsbruggen *et al.*, 2004 ▸; Phonsri *et al.*, 2017 ▸; Powell *et al.*, 2014 ▸, 2015 ▸, 2020 ▸, 2022 ▸; Powell, 2016 ▸; Yemeli Tido, 2010 ▸; Zelentsov *et al.*, 1973 ▸; Ryabova *et al.*, 1978 ▸, 1981*a* ▸,*b* ▸, 1982 ▸; Floquet *et al.*, 2003 ▸, 2006 ▸, 2009 ▸; Li *et al.*, 2013 ▸, 2016 ▸).

Our research demonstrated that the electronic state of an Fe^III^ ion surrounded by two such tridentate *O*,*N*,*S*-thio­semi­car­ba­zon­ate ligands depends on the substituents and degree of deprotonation of the *R*-salicyl­aldehyde 4*R*′-thio­semi­car­ba­zone ligands, the identity of the counter-ion and the nature and degree of solvation (Powell *et al.*, 2014 ▸, 2015 ▸, 2020 ▸, 2022 ▸; Powell, 2016 ▸; Yemeli Tido, 2010 ▸).

In fact, in solution, the free *R*-salicyl­aldehyde 4*R*′-thio­semicarbazone ligand (H_2_ *L*) exists in two tautomeric forms, *i.e.* the thione and thiol forms, as illustrated in Scheme 1. Moreover, the ligand may also be present in its neutral, anionic or dianionic form. We established that the formation of a particular type of Fe^III^ com­plex unit, *i.e.* neutral, monocationic or monoanionic, can be achieved by tuning the degree of deprotonation of the ligand through pH variation of the reaction solution during the synthesis (Powell *et al.*, 2014 ▸, 2015 ▸, 2020 ▸, 2022 ▸; Powell, 2016 ▸; Yemeli Tido, 2010 ▸; Floquet *et al.*, 2009 ▸). 
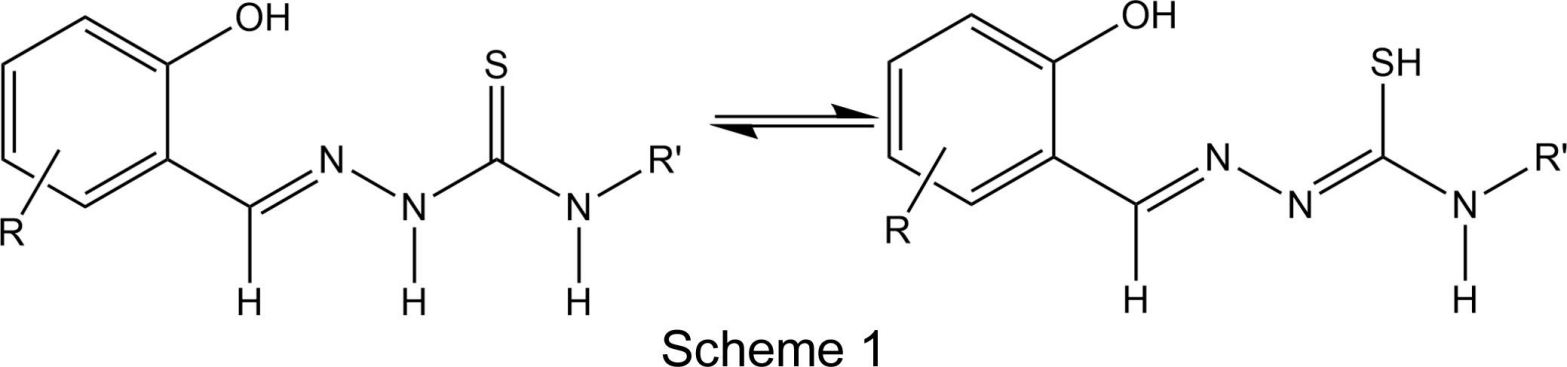


We have thus been particularly proficient in preparing anionic Fe^III^ com­plexes of the general formula (cation^+^)[Fe(*L* ^2−^)_2_]·*x*(solvent), such as Cs[Fe(3-OEt-thsa-Me)_2_]·CH_3_OH, containing 3-eth­oxy­salicyl­aldehyde methyl­thio­semi­car­ba­zon­ate(2−) (Powell *et al.*, 2014 ▸), Cs[Fe(5-Br-thsa)_2_] containing 5-bromo­salicyl­aldehyde thio­semi­car­ba­zon­ate(2−) (Powell *et al.*, 2015 ▸) and NH_4_[Fe(thsa)_2_] containing salicyl­aldehyde thio­semi­car­ba­zon­ate(2−) (Powell *et al.*, 2020 ▸). In all of these com­pounds, Fe^III^ exhibits the low-spin state.

Here we report a novel Fe^III^ com­pound of this family, namely, di­methyl­ammonium bis­[3-eth­oxy­salicyl­aldehyde thio­semi­car­ba­zon­ato(2–)-κ^3^ *O* ^2^,*N* ^1^,*S*]ferrate(III), [(CH_3_)_2_NH_2_][Fe(3-OEt-thsa)_2_], (I) (see Scheme 2), containing two dianionic tri­dentate ligands, *i.e.* 3-eth­oxy­salicyl­aldehyde thio­semi­car­ba­zon­ate(2−), whose structure was determined at 100 K and confirmed that Fe^III^ is in the high-spin state.

## Experimental

### Spectroscopic and magnetic measurements

A room-tem­per­ature IR spectrum of 3-eth­oxy­salicyl­alde­hyde thio­semicarbazone within the range 4000–400 cm^−1^ was recorded on a PerkinElmer FT–IR spectrometer Spectrum RXI using KBr pellets. IR spectroscopic measurements of (I) within the range 4000–600 cm^−1^ were carried out at room tem­per­ature using an ATR (attenuated total reflectance) PerkinElmer FT–IR Frontier spectrometer. 
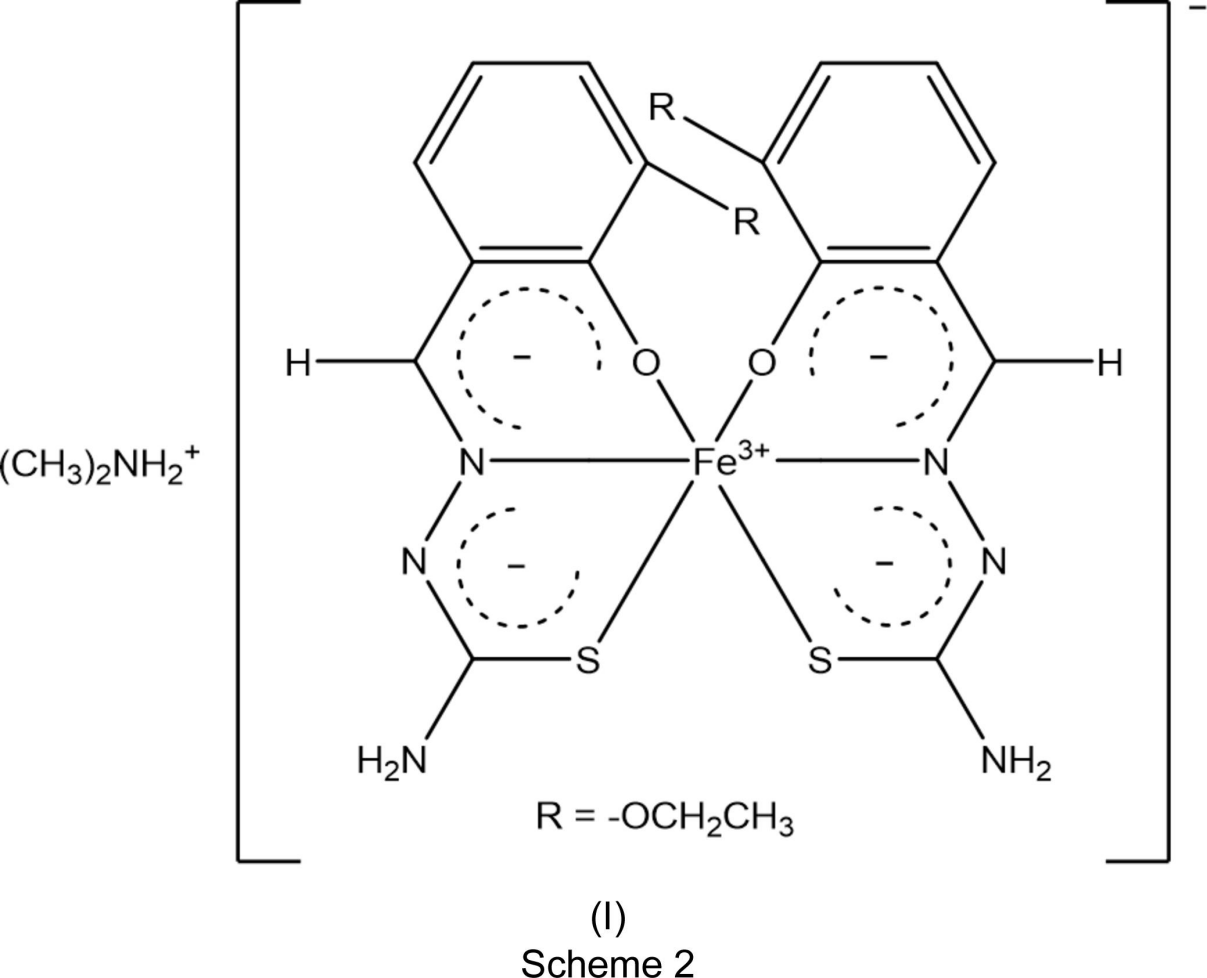


^1^H and ^13^C NMR spectra were recorded in DMSO-*d*_6_ (dimethyl sulfoxide) using a Bruker cryomagnet BZH 300/52 spectrometer (300 MHz), with the recorded chemical shifts in δ (in parts per million) relative to an inter­nal standard of tetra­methyl­silane (TMS).

Measurements of direct current (dc) magnetic susceptibility, χ_M_, *versus* tem­per­ature, *T*, were conducted between 5 and 320 K, heating and cooling at a rate of 2 K min^−1^ in an applied field, μ_0_ *H*, of 0.1 T using a Quantum Design MPMS-5S super­conducting quantum inter­ference device (SQUID) magnetometer. The SQUID magnetometer was calibrated using a standard palladium sample. The background due to the sample holder and the diamagnetic signal of the sample, estimated using Pascal’s constants (Bain *et al.*, 2008 ▸), was subtracted from the measured molar magnetic susceptibility χ_M_.

### Synthesis

The synthesis of 3-eth­oxy­salicyl­aldehyde thio­semi­car­ba­zone (H_2_-3-OEt-thsa) was carried out according to the general procedure described by Yemeli Tido (2010 ▸) (yield: 11.14 g, 46.55 mmol, 95.0%; m.p. 224 °C). H_2_-3-OEt-thsa is soluble in methanol, ethanol, acetone and DMSO. ^1^H NMR (300 MHz, DMSO-*d*_6_): δ (ppm) 11.39 (1H, *s*, OH), 9.02 (1H, *s*, S=C—NH), 8.40 (1H, *s*, N=C—H), 7.90–8.13 (2H, *m*, S=C—NH_2_), 6.72–7.50 (aromatic 3H, *m*, C—H), 4.05 (2H, *q*, O—CH_2_), 1.35 (3H, *t*, O—C—CH_3_). ^13^C NMR (300 MHz, DMSO-*d*_6_): δ (ppm) 182.8 (C=S), 147.4, 146.7 (C—O), 140.1 (C=N), 119.5, 118.7, 114.5 (C aromatic), 64.6 (C—N), 74.0 (O—CH_2_), 15.1 (O—C—CH_3_). IR (cm^−1^, KBr): 3400 (νOH), 3169 (νNH), 3249 (νNH_2_), 2935 (νCH_3_), 2896 (νCH_2_), 1618 (νC=N), 1535–1600 (νC=C), 1270 (νC—N), 1167 (νC=S).

The synthesis of [(CH_3_)_2_NH_2_][Fe(3-OEt-thsa)_2_], (I), was carried out as follows: Fe(NO_3_)_3_·9H_2_O (1.0 mmol, 0.40 g) was dissolved in water (10 ml). The ligand H_2_-3-OEt-thsa (2.0 mmol, 0.46 g) was dissolved in methanol (60 ml) with the addition of di­methyl­amine, 40 wt% in water (10 mmol, 0.51 ml). To this mixture, the Fe^III^ salt solution was added dropwise with constant stirring. The resulting dark-green solution was stirred and heated to 80 °C for approximately 10 min. The solution was then allowed to stand at room tem­per­ature until crystals had formed. The dark-green microcrystals were isolated by filtration and dried (yield: 0.30 g, 0.52 mmol, 52.0%). IR (cm^−1^, ATR): 3436, 3414 (νNH), 3265, 3098 (νNH_2_), 3012 (νCH_3_), 2971 (νCH_2_), 1614, 1586 (νC=N), 1570–1541 (νC=C ring), 1238 (νC—O), 1215 (νN—N), 1078 (νC—N), 736 (νC—S).

### Refinement

Crystal data, data collection and structure refinement details are summarized in Table 1 ▸. The H atoms of terminal amine atoms N103 and N3 were located in difference Fourier maps and refined with restrained N—H distances of 0.86 (2) Å and with *U*_iso_(H) = 1.2*U*_eq_(N). The remaining H atoms were included in the refinement in calculated positions and treated as riding on their parent atoms, with N—H distances of 0.91 Å and *U*_iso_(H) = 1.2*U*_eq_(N) for the amine N atom of the cation, C—H distances of 0.95 Å and *U*_iso_(H) = 1.2*U*_eq_(C) for aryl (–CH=) H atoms, C—H distances of 0.99 Å and *U*_iso_(H) = 1.2*U*_eq_(C) for secondary (–CH_2_–) H atoms, and C—H dis­tances of 0.98 Å and *U*_iso_(H) = 1.5*U*_eq_(C) for methyl (–CH_3_) H atoms.

## Results and discussion

In solution, the free ligand, *i.e.* 3-eth­oxy­salicyl­aldehyde thio­semicarbazone (H_2_ *L*), exists in two tautomeric forms, the thione and the thiol form, as illustrated in Scheme 1. Consequently, in Fe^III^ com­pounds, the ligand may be present as either one of the possible tautomers, and may be neutral, anionic or dianionic. Referring to the thiol tautomer, neutral H_2_ *L* has H atoms located on the phenol O atom and the thiol S atom. The first deprotonation step involving the phenol group results in the formation of 3-eth­oxy­salicyl­aldehyde thio­semicarbazone(1−) (abbreviated as H*L* ^−^). Subsequent deprotonation yields 3-eth­oxy­salicyl­aldehyde thio­semi­car­ba­zon­ate(2−) (abbreviated as *L* ^2−^).

**Figure 1:**
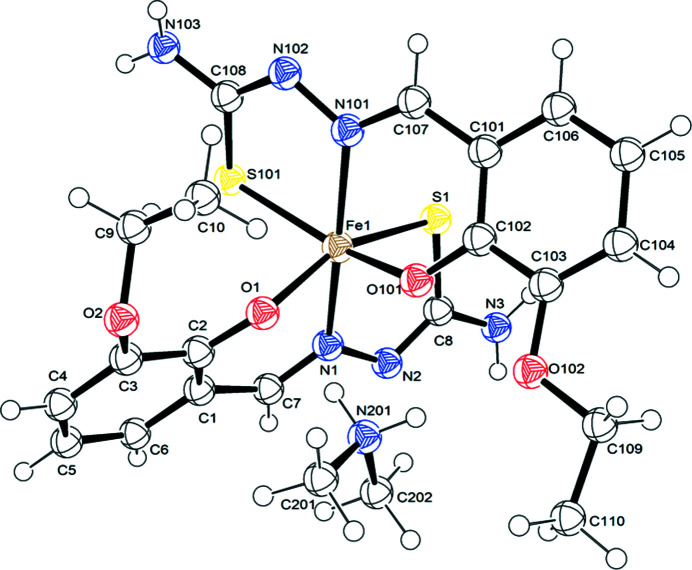
The mol­ecular structure and atom-numbering scheme for (I). Displacement ellipsoids are drawn at the 50% probability level.

The structure of di­methyl­ammonium bis­[3-eth­oxy­salicyl­aldehyde thio­semi­car­ba­zon­ato(2−)-κ^3^ *O* ^2^,*N* ^1^,*S*]ferrate(III), (I) (Fig. 1 ▸), was determined at 100 K. Compound (I) crystallized in the monoclinic space group *P*2_1_/*n*, with *Z* = 4. The asymmetric unit consists of one formula unit, [(CH_3_)_2_NH_2_][Fe(3-OEt-thsa)_2_], with no atom on a special position. The Fe^III^ cation is coordinated by the thiol­ate S, phenolate O and imine N atoms of each of the two dianionic *O*,*N*,*S*-tridentate *L* ^2−^ ligands. The donor atoms of the ligands are situated in two perpendicular planes, with the O and S atoms in *cis* positions, and mutually *trans* N atoms. Selected geometric parameters are listed in Table 2 ▸.

The Fe^III^O_2_N_2_S_2_ coordination sphere exhibits a distorted octa­hedral geometry, as evidenced by the bond angles of the Fe atom and the ligand donor atoms (*vide infra*). X-ray structural data of similar Fe^III^–bis­(ligand) com­pounds con­taining two dianionic thio­semi­car­ba­zon­ate(2−) ligands show that the Fe—S, Fe—O and Fe—N bond lengths are in the ranges 2.23–2.31, 1.93–1.95 and 1.88–1.96 Å, respectively, for low-spin Fe^III^ com­pounds, and in the ranges 2.40–2.44, 1.96–1.99 and 2.05–2.15 Å, respectively, for the corresponding high-spin Fe^III^ com­pounds (van Koningsbruggen *et al.*, 2004 ▸). The bond lengths involving the Fe atom and the donor atoms in (I) correspond with Fe^III^ being in the high-spin state at 100 K.

Variable-tem­per­ature magnetic susceptibility measurements (5–320 K) confirm that the Fe^III^ ion in (I) is indeed in the high-spin state over this tem­per­ature range (Powell, 2016 ▸). High-spin Fe^III^ has also been evidenced in the related Cs[Fe(thsa)_2_] com­pound at 103 (and 298 K) (Ryabova *et al.*, 1981*a* ▸). It is significant to note that the Fe—O distances seem to be less sensitive to the change in Fe^III^ spin state than the Fe—N and Fe—S distances, which may be related to the π-acceptor capability of the N- and S-donor atoms as opposed to the π-donor capability of the O-donor atoms. This is of particular significance when Fe^III^ is in the low-spin state, as increased π backbonding will lead to com­paratively more pronounced shortening of the Fe—N and Fe—S bonds than of the Fe—O bonds (Powell *et al.*, 2014 ▸).

Furthermore, the spin state of the Fe^III^ cation can be related to the bond angles of the FeO_2_N_2_S_2_ coordination core. An analysis of the bond angles involving the opposite ligand donor atoms at 100 K is very enlightening, as it shows that the octa­hedral geometry of the present high-spin Fe^III^ com­pound, with O1—Fe1—S1 = 158.48 (5)°, O101—Fe1—S101 = 158.89 (5)° and N1—Fe1—N101 = 167.63 (7)°, is considerably less regular than that of the low-spin com­pound Cs[Fe(3-OEt-thsa-Me)_2_]·CH_3_OH, with the bond angles S11—Fe—O11 = 177.83 (14)°, S21—Fe—O21 = 178.01 (13)° and N11—Fe—N21 = 178.9 (2)° (Powell *et al.*, 2014 ▸), which are closer to 180°. This is in agreement with the low-spin Fe^III^ ion adopting a more regular octa­hedral geometry than the high-spin Fe^III^ ion (van Koningsbruggen *et al.*, 2004 ▸).

The ligands have been found to be in the dianionic form as no H atoms were located on the phenolate O (O1 and O101) or the thiol­ate S (S1 and S101) atoms. The charge of the two *L* ^2−^ ligands is balanced by the presence of the monovalent di­methyl­ammonium cation together with the trivalent iron cation. The tridentate ligands of the present com­pound are coordinated to the Fe^III^ cation by the thiol­ate S, phenolate O and imine N atoms, forming six- and five-mem­bered chelate rings. The six-mem­bered chelate ring involves a significantly less restricted bite angle [O1—Fe—N1 = 82.17 (7)° and O101—Fe—N101 = 84.03 (7)°] than the five-mem­bered chelate ring [S1—Fe—N1 = 78.45 (5)° and S101—Fe—N101 = 78.93 (5)°]. The r.m.s. deviations from their least-squares plane of atoms of the six-mem­bered chelate ring of both coordinated ligands are 0.197 and 0.177 Å for Fe1/N11/C17/C11/C12/O11 and Fe1/N101/C107/C101/C102/O101, respectively, and the corresponding values for the five-mem­bered chelate rings are 0.129 and 0.102 Å for Fe1/N11/C12/C18/S11 and Fe1/N101/C102/C108/S101, respectively. It appears that the metal chelate rings deviate slightly from the ideal planar structure. Furthermore, the O—Fe—N and S—Fe—N bite angles of the six- and five-mem­bered chelates are deficient by *ca* 38 and 30°, respectively, com­pared to the angle at the vertex of a regular hexa­gon (120°) or penta­gon (108°), respectively. In com­parison to other (cation^+^)[Fe(*L* ^2−^)_2_]·*x*(solvent) com­pounds of related ligands (Powell *et al.*, 2014 ▸, 2015 ▸, 2020 ▸), the deficiency of the bite angle in both the six- and five-mem­bered chelate rings is larger than expected, though it has been recognized that these other Fe^III^ bis­(ligand) com­pounds contain Fe^III^ in the low-spin state, whereas the present com­pound contains Fe^III^ in the high-spin state. Consequently, (I) displays longer Fe^III^–donor atom bond lengths, which are associated with more restricted bite angles. Moreover, the remaining bond angles involving each six-mem­bered chelate ring (Table 2 ▸) are, as expected, within *ca* 5° of the value of 125°. However, the C—S—Fe bond angles involving each five-mem­bered chelate ring are only about 95°, providing an additional deficiency of 13°. The additional deficiency can be offset by increasing the other bond angles within this five-mem­bered chelate ring to *ca* 120°. It has been found that the N—N—C angles are <120° and the N—C—S angles are >120°; these values suggest *sp* ^2^ hybridization at the C and N atoms.

The stability of the Fe^III^ com­plex is further enhanced by the high degree of electron delocalization throughout the chelated ligands, which is evident from the geometric parameters. The C—S, C—N and N—N bond lengths of (I) show characteristics of a bond order between 1 (*i.e.* single bond) and 2 (*i.e.* double bond). The C8—S1 bond length of 1.746 (3) Å and the C108—S101 bond length of 1.752 (2) Å suggest partial electron delocalization of these C—S bonds. This feature has also been found in the structure of the related high-spin Fe^III^ com­pound Cs[Fe(thsa)_2_] at 103 K (Ryabova *et al.*, 1981*a* ▸), in which the C—S bond lengths of 1.749 (9) and 1.761 (9) Å are indicative of partial electron delocalization.

In addition, the electron delocalization within each of the *O*,*N*,*S*-tridentate ligands is confirmed by a bond order larger than 1 for the C—N bond involving the deprotonated hydrazinic N atom, which is inferred from the lengths for the C7—N1 and C107—N101 bonds in (I) at 100 K of 1.301 (3) and 1.301 (3) Å, respectively, which correspond to the C—N bond lengths of 1.314 (10) and 1.303 (11) Å, respectively, for Cs[Fe(thsa)_2_] at 103 K (Ryabova *et al.*, 1981*a* ▸).

Moreover, the N—N bond lengths of (I) at 100 K are N1—N2 of 1.395 (2) Å and N101—N102 of 1.399 (3) Å, which indicates partial electron delocalization within the five-mem­bered chelate ring.

**Figure 2:**
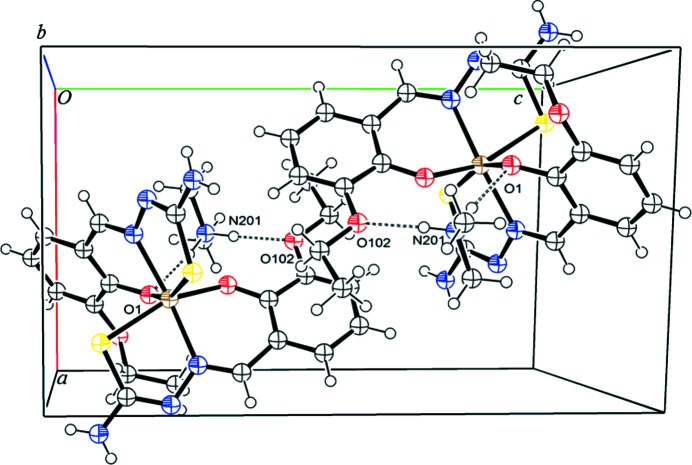
The unit cell of (I), with displacement ellipsoids drawn at the 50% probability level.

The hydro­gen-bonding inter­actions of (I), identified using the default parameters of *OLEX2* (Dolomanov *et al.*, 2009 ▸), are listed in Table 3 ▸ and displayed in Fig. 2 ▸. The N atom of the di­methyl­ammonium cation forms two hydro­gen bonds: one contact is formed with the phenolate O atom of one ligand, whereas the second contact is formed with the eth­oxy O atom of the sali­cyl­aldehyde moiety of the other ligand. The N201—H20*A*⋯O102 and N201—H20*B*⋯O1 contacts form an intra­molecular hydro­gen-bonded ring system, giving rise to an 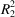 (9) ring (Bernstein *et al.*, 1995 ▸).

**Figure 3:**
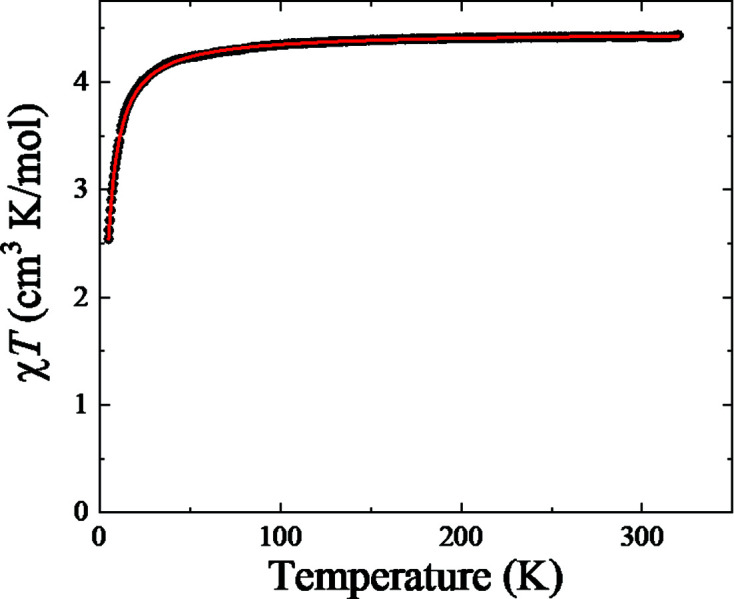
χ_M_ *T versus T* for (I). The data were measured while cooling at a rate of 2 K min^−1^ in an applied field μ_0_ *H* of 0.1 T.

**Figure 4:**
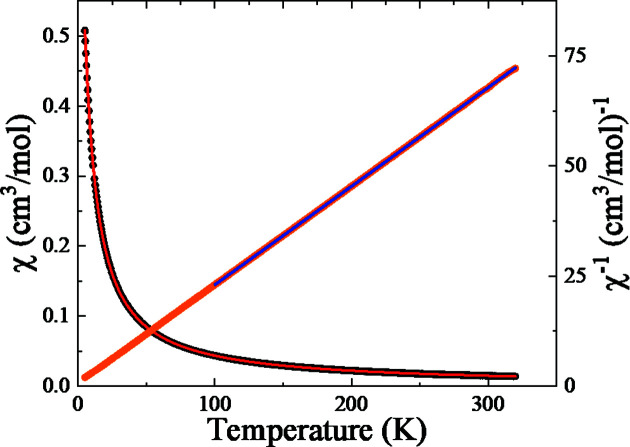
Temperature dependence of the molar magnetic susceptibility, χ_M_, for (I). The solid red lines show fits to the data using Equation (1), with *D* = 0.83 (1) cm^−1^ and *g* = 2. The blue line shows a fit of χ_M_^−1^(*T*) above 100 K using a Curie–Weiss law.

Magnetic susceptibility *versus* tem­per­ature measurements for (I) were carried out to investigate the spin state of the Fe^III^ ion. The data collected on heating and cooling coincide over the tem­per­ature range studied. The tem­per­ature dependence of χ_M_ *T* collected on cooling between 320 and 5 K is displayed in Fig. 3 ▸. Above 100 K, χ_M_ *T* is tem­per­ature independent with a value of 4.41 (1) cm^3^ K mol^−1^ [5.94 (1) µ_B_/Fe]. This is just above the expected value of 4.38 cm^3^ K mol^−1^ (5.92 µ_B_/Fe) for Fe^III^ in its high-spin (*S* = 5/2) state with an electronic *g* factor of 2. χ_M_^−1^(*T*) is linear in *T* and a fit to a Curie–Weiss law between 100 and 320 K shown in Fig. 4 ▸ gives a Weiss tem­per­ature of −3.3 (1) K and an effective moment of 6.00 (1) µ_B_/Fe.

χ_M_ *T* drops rapidly below 100 K. This may be due to weak (anti­ferro)magnetic inter­actions between neighbouring spins or may reflect a splitting of the *S* = 5/2 state (O’Connor, 1982 ▸). Studies using aligned single crystals are needed to differentiate between these possibilities. For splitting, the spin Hamiltonian can be written as *H*_S_ = *H*_CEF_ + *H_z_*, where the crystalline electric field (CEF) term *H*_CEF_ = *D*[*S_z_* ^2^ − *S*(*S *+ 1)/3] + *E*(*S_x_* ^2^ – *S_y_* ^2^), with *D* and *E* being the axial and rhombic zero-field splitting, respectively. The ^6^ *S* high-spin state is split into three Kramers doublets. For *E* = 0, the doublets are separated by 2*D* and 6*D* from the lowest energy doublet. The Zeeman energy *H_z_* = *g*μ_B_ *HS_z_* and the molar susceptibility with a field along *z* is

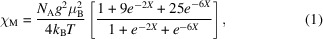

where *X* = *D*/*k*_B_ *T*, *N*_A_ is Avogadro’s number and *k*_B_ is the Boltzmann constant (O’Connor, 1982 ▸). A fit gives *D* = 0.83 (1) cm^−1^ with *g* = 2. *D* is in the range expected for high-spin Fe^III^ (Chen *et al.*, 2002 ▸; Yemeli Tido *et al.*, 2007 ▸). Fits with a finite *E* expected for a system with a rhombic distortion are possible, *cf*. Chen *et al.* (2002 ▸), but these require a knowledge of the ratio λ = *E*/*D* from other studies, such as electron para­magnetic resonance (EPR) spectroscopy.

It is of inter­est to com­pare the two Fe^III^ com­pounds that have so far been reported to contain the 3-eth­oxy­salicyl­aldehyde 4-*R*′-thio­semi­car­ba­zon­ate(2−) dianion. In Cs[Fe(3-OEt-thsa-Me)_2_]·CH_3_OH (Powell *et al.*, 2014 ▸), Fe^III^ is low spin, whereas in the present [(CH_3_)_2_NH_2_][Fe(3-OEt-thsa)_2_] com­pound, (I), the metal ion adopts the high-spin state. The differences between the two com­pounds further involve: (i) the relative size of the *R*′ substituent on the terminal N atom of the thio­semicarbazide moiety, as (I) contains a H atom, whereas Cs[Fe(3-OEt-thsa-Me)_2_]·CH_3_OH (Powell *et al.*, 2014 ▸) contains a methyl substituent; (ii) the difference in the size and inter­molecular inter­actions involving the associated outer-sphere monovalent cation, *i.e.* (CH_3_)_2_NH_2_^+^ *versus* Cs^+^; and (iii) the presence of a methanol solvent mol­ecule in the crystal lattice of Cs­[Fe(3-OEt-thsa-Me)_2_]·CH_3_OH (Powell *et al.*, 2014 ▸). These differences are associated with (I) forming intra­molecular ring systems through hydro­gen bonds (*vide supra*), whereas Cs­[Fe(3-OEt-thsa-Me)_2_]·CH_3_OH forms inter­mole­cular hydro­gen-bonded ring systems which link neighbouring Fe^III^ entities. These factors determine the arrangement of the Fe^III^ entities within the unit cell, which is further characterized by the space group *P*2_1_/*n*, with *Z* = 4 and *V* = 2576.35 (17) Å^3^ for (I), with a volume of 644.09 Å^3^ per high-spin Fe^III^ formula unit, and the space group *P* 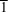 , with *Z* = 2 and *V* = 1369.5 (8) Å^3^ for Cs[Fe(3-OEt-thsa-Me)_2_]·CH_3_OH, with a volume of 684.75 Å^3^ per low-spin Fe^III^ formula unit (Powell *et al.*, 2014 ▸); hence the volume increase associated with Fe^III^ being low-spin com­pared to high-spin is more than offset by the differences in substituents, com­position and crystal packing.

Evidently, the intricate inter­play between the variation in cation, ligand substituents and associated solvent mol­ecules affects the crystal packing of com­pounds of this class of (cation^+^)[Fe(*L* ^2−^)_2_]·*x*(solvent) materials and allows for a variation of the spin state of Fe^III^, with some members dis­playing tem­per­ature-dependent spin-crossover behaviour (van Koningsbruggen *et al.*, 2004 ▸; Powell, 2016 ▸). Further studies by our group will additionally focus on tuning the spin state of Fe^III^ by varying the degree of deprotonation of the ligand.

## Supplementary Material

Crystal structure: contains datablock(s) I, global. DOI: 10.1107/S2053229622011597/jx3075sup1.cif

Structure factors: contains datablock(s) I. DOI: 10.1107/S2053229622011597/jx3075Isup2.hkl

CCDC reference: 2223775

## Figures and Tables

**Table 1:** Experimental details Diffractometer: Rigaku AFC12 goniometer equipped with an enhanced sensitivity (HG) Saturn724+ detector mounted at the window of an FR-E+ SuperBright molybdenum rotating anode generator with VHF Varimax optics (70 µm focus).

Crystal data	
Chemical formula	(C_2_H_8_N)[Fe(C_10_H_11_N_3_O_2_S)_2_]
*M*_r_	576.50
Crystal system, space group	Monoclinic, *P*2_1_/*n*
Temperature (K)	100
*a*, *b*, *c* (Å)	9.4359 (3), 16.0265 (5), 17.2333 (7)
β (°)	98.668 (4)
*V* (Å^3^)	2576.35 (17)
*Z*	4
Radiation type	Mo *K*α
μ (mm^−1^)	0.79
Crystal size (mm)	0.08 × 0.05 × 0.01

Data collection	
Absorption correction	Multi-scan (*CrysAlis PRO*; Agilent, 2014 ▸)
*T*_min_, *T*_max_	0.661, 1.000
No. of measured, independent and observed [*I* > 2σ(*I*)] reflections	16969, 5903, 4415
*R*_int_	0.055
(sin θ/λ)_max_ (Å^−1^)	0.649

Refinement	
*R*[*F* ^2^ > 2σ(*F* ^2^)], *wR*(*F* ^2^), *S*	0.044, 0.096, 1.02
No. of reflections	5903
No. of parameters	341
No. of restraints	4
H-atom treatment	H atoms treated by a mixture of independent and constrained refinement
Δρ_max_, Δρ_min_ (e Å^−3^)	0.38, −0.38

Computer programs: *CrystalClear-SM Expert* (Rigaku, 2013 ▸), *CrysAlis PRO* (Agilent, 2014 ▸), *SUPERFLIP* (Palatinus & Chapuis, 2007 ▸; Palatinus & van der Lee, 2008 ▸), *SHELXL2014* (Sheldrick, 2015 ▸), *OLEX2* (Dolomanov *et al.*, 2009 ▸) and *ORTEP-3 for Windows* (Farrugia, 2012 ▸).

**Table 2:** Selected geometric parameters (Å, °)

Fe1—S1	2.4320 (6)	Fe1—O101	1.9595 (16)
Fe1—S101	2.4389 (7)	Fe1—N1	2.167 (2)
Fe1—O1	1.9806 (16)	Fe1—N101	2.131 (2)
			
S1—Fe1—S101	98.98 (2)	C108—S101—Fe1	95.71 (8)
O1—Fe1—S1	158.48 (5)	C2—O1—Fe1	127.41 (14)
O1—Fe1—S101	91.30 (5)	C102—O101—Fe1	130.25 (16)
O1—Fe1—N1	82.17 (7)	C7—N1—Fe1	124.80 (15)
O1—Fe1—N101	107.31 (7)	C8—N2—N1	113.96 (19)
O101—Fe1—S1	94.31 (5)	C107—N101—Fe1	123.56 (16)
O101—Fe1—S101	158.89 (5)	C108—N102—N101	114.09 (19)
O101—Fe1—O1	81.91 (7)	C2—C1—C7	121.2 (2)
O101—Fe1—N1	105.56 (7)	O1—C2—C1	122.4 (2)
O101—Fe1—N101	84.03 (7)	N1—C7—C1	125.3 (2)
N1—Fe1—S1	78.45 (5)	N2—C8—S1	125.75 (17)
N1—Fe1—S101	93.18 (5)	C102—C101—C107	121.2 (2)
N101—Fe1—S1	93.26 (5)	O101—C102—C101	123.1 (2)
N101—Fe1—S101	78.93 (5)	N101—C107—C101	125.9 (2)
N101—Fe1—N1	167.63 (7)	N102—C108—S101	125.65 (18)
C8—S1—Fe1	95.87 (8)		

**Table 3:** Hydrogen-bond geometry (Å, °)

*D*—H⋯*A*	*D*—H	H⋯*A*	*D*⋯*A*	*D*—H⋯*A*
N201—H20*A*⋯O102	0.91	1.97	2.877 (3)	174
N201—H20*B*⋯O1	0.91	1.86	2.766 (3)	173
